# Permeation Studies across Symmetric and Asymmetric Membranes in Microdroplet Arrays

**DOI:** 10.1021/acs.analchem.0c04939

**Published:** 2021-03-15

**Authors:** Simon Bachler, Marion Ort, Stefanie D. Krämer, Petra S. Dittrich

**Affiliations:** † Department of Biosystems Science and Engineering, ETH Zurich, 4058 Basel, Switzerland; ‡ Institute of Pharmaceutical Sciences, Department of Chemistry and Applied Biosciences, ETH Zurich, Zürich 8093, Switzerland

## Abstract

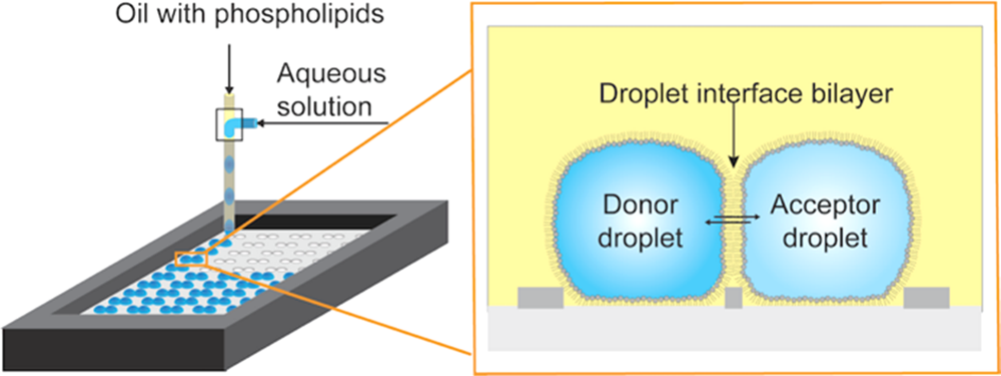

We investigated the permeation of molecules across lipid membranes on an open microfluidic platform. An array of droplet pairs was created by spotting aqueous droplets, dispersed in a lipid oil solution, onto a plate with cavities surrounded by a hydrophobic substrate. Droplets in two adjacent cavities come in contact and form an artificial lipid bilayer, called a droplet interface bilayer (DIB). The method allows for monitoring permeation of fluorescently tagged compounds from a donor droplet to an acceptor droplet. A mathematical model was applied to describe the kinetics and determine the permeation coefficient. We also demonstrate that permeation kinetics can be followed over a series of droplets, all connected via DIBs. Moreover, by changing the lipid oil composition after spotting donor droplets, we were able to create asymmetric membranes that we used to mimic the asymmetry of the cellular plasma membrane. Finally, we developed a protocol to separate and extract the droplets for label-free analysis of permeating compounds by liquid chromatography–mass spectrometry. Our versatile platform has the potential to become a new tool for the screening of drug membrane permeability in the future.

## Introduction

Most pharmaceutical small-molecule drugs are primarily administered orally and mainly absorbed in the small intestine.^1^ As the transcellular route is the most relevant for the absorption of these drugs, assays for predicting membrane permeability play an important role during the drug discovery and development process.^2−5^ A commonly used cell-free *in vitro* permeability testing technique is the parallel artificial membrane permeability assay (PAMPA).^3,4,6,7^ In this method, two-layered multiwell plates are used to measure permeation through barriers formed between the top and bottom wells. PAMPA is suitable for predicting purely lipoidal permeation; this is not possible in cell-based assays, in which carrier-mediated and lipoidal permeation coexist.^2−4,6,7^ In PAMPA, the barriers formed between the donor and acceptor wells consist of porous filters with a typical thickness of ∼10 to 100 μm soaked in a mixture of lipid and hydrocarbon oil or pure hydrocarbon oil.^3,4,7^ Hence, the barriers in PAMPA are much thicker than a lipid bilayer (∼5 nm), and their structure is dissimilar to actual biological membranes. Furthermore, diffusion through such a thick barrier, measured by concentration changes in the acceptor and donor wells with volumes of several hundred microliters, leads to assay times in the range of 2–18 h, which can limit the throughput.^4,7^ Common alternatives are *in vitro* cell-based permeation assays, using Caco-2 and Madin-Darby canine kidney (MDCK) cellular monolayers.^2,4^ However, cellular monolayers are not always well suited for a systematic and mechanistic investigation of drug absorption and require a laborious cell culture.

Recently, microfluidic methods have been introduced to capture cell mimicking vesicles for permeation studies^8−11^ as well as to create on-chip artificial cell membranes.^12,13^ A significant advantage of microfluidic devices compared to traditional laboratory methods is the small size and, associated with this, the small sample volumes.^14,15^ In this regard, droplet-based microfluidics is particularly intriguing as it enables the generation of nano- to picoliter-sized aqueous droplets continuously and at a high frequency. The aqueous phase is injected into a carrier fluid that is not miscible with water; often, surfactants are used for stabilization.^16^ When lipids are added to the oil phase, they form a monolayer at the droplet–oil interface. Two adjacent droplets that contact each other form the so-called droplet interface bilayer (DIB).^4,12,13,17^ The DIBs allow translocation of membrane-permeable compounds from one donor droplet to the acceptor droplets by passive diffusion. Such droplet systems are therefore interesting approaches for permeation studies, however, require fluorescently labeled compounds^18^ or fluorogenic assays to visualize permeation of weakly basic or acidic compounds.^8^ Fluorescence spectroscopy is sufficiently sensitive and adaptable to the small volumes, but as the label—often a hydrophobic fluorophore—may influence the permeability,^10,19^ label-free detection would be favorable.^20,21^ In this context, open platforms with so-called static droplet arrays were introduced for miniaturizing biological and chemical processes and reactions, and they proved particularly versatile for implementing analytical methods beyond optical microscopy, such as mass spectrometry,^22−24^ but were not used for permeability assays so far.

Here, we introduce a microfluidic method that combines the advantages of miniaturization and enables monitoring of fluorescent and nonfluorescent compounds. Our platform facilitates automated and precise positioning of droplet pairs to create DIBs. Permeation of compounds occurs along the concentration gradient from a donor droplet to an acceptor droplet that initially contains no drug. We precisely describe this process using a mathematical model and derive the permeability constants for fluorescent compounds. We also demonstrate the possibility to generate asymmetric lipid bilayers, thereby reflecting cell membranes in a more realistic model.^25^ Moreover, in contrast to previous approaches to form DIBs, we can uptake individual nanoliter droplets and developed a protocol to analyze them by liquid chromatography–mass spectrometry (LC–MS). We apply this new established method for label-free detection of a model permeant.

## Materials and Methods

### Assay Preparation

The lipid-out approach was used to add phospholipids to the water/oil interface, i.e., lipids were dissolved in the oil phase. We purchased 1,2-dioleoyl-*sn*-glycero-3-phosphocholine (DOPC) and 1,2-dioleoyl-*sn*-glycero-3-phospho-L-serine (DOPS) as solutions in chloroform from Avanti Polar Lipids (Alabaster, AL, U.S.A.). We placed the required lipids in pear-shaped flasks and removed the chloroform with a rotary evaporator (Büchi Labortechnik AG, Flawil, Switzerland) forming a film. The lipid film was dissolved in a 1:1 (v/v) mix of hexadecane (reagent plus grade, Sigma–Aldrich) and squalane (Sigma–Aldrich) in an ultrasonication bath at 50 °C for ∼30 min. The final phospholipid concentration in oil was 0.625 mM for donor droplets and 1.25 mM for acceptor droplets. Lower concentrations of phospholipids resulted in the formation of unstable DIBs (often droplet pair fused), while excessive phospholipids in the oil led to extraction of compounds into the oil, presumably by formation of inverse micelles.

We used either 100% DOPC or 30% DOPC and 70% DOPS mixtures (mole percentage). We filtrated the phospholipid-oil solution before use (0.45 μm pore size, RC 4 Male Luer Slip Minisart filters, Huberlab, Switzerland).

All aqueous solutions were prepared in LC–MS grade water (Fisher Scientific, Loughborough, U.K.). We used as buffers 20 mM 2-morpholinoethanesulfonic acid (MES, pH 6.0, Alfa Aesar), 20 mM phosphate buffer (pH 7.0, Acros Organics), 50 mM 4-(2-hydroxyethyl)-1-piperazineethanesulfonic acid (HEPES, pH 7.4, gibco, Paisley, U.K.), or 20 mM tris(hydroxymethyl)aminomethane (Tris, pH 8.0, VWR). The following fluorophores were dissolved in buffer: 50 μM rhodamine 6G (laser grade, Acros Organics), 50 μM fluorescein (Honeywell Fluka, Seelze, Germany), ∼50 μM PEG4-NBD, and 50 μM calcein (Sigma–Aldrich). PEG4-NBD was synthesized from succinimidyl 6-(*N*-(7-nitrobenz-2-oxa-1,3-diazol-4-yl)amino)hexanoate (NBD NHS-ester, Molecular Probes Life Technologies, Eugene, OR, U.S.A.) and amine-terminated poly(ethylene glycol)-4 alcohol (Amino-PEG4-OH, Quanta Biodesign, Plain City, OH, U.S.A.).^26^ A 1:1 molar ratio of the NBD NHS-ester and Amino-PEG4-OH was reacted in a 10:1 (v/v) solution of chloroform (Sigma–Aldrich) and triethylamine (Brenntag Schweizerhall, Basel, Switzerland). The reaction was held at 45 °C for 2 h, followed by 22 °C for 12 h. Afterward, the reaction products were separated with a preparative liquid chromatography system (Prep 150 LC system, Waters). The mass of the product PEG4-NBD was confirmed with an LC–MS system (Ultimate 3000 MSQ, Dionex).

### Fabrication of the Microarray Plates

We used a previously developed protocol for fabricating microarray plates with cavities.^24^ In brief, a 4 inch borofloat glass wafer was cleaned by oxygen plasma and subsequently spin-coated with SU-8 3025 (MicroChem, Westborough, MA, U.S.A.) to obtain an approximately 35 μm-high layer of photoresist. The wafer was soft baked at 65 °C for 2 min and 95 °C for 12 min. Afterward, we exposed the photoresist to a UV light source through a foil mask (i-line illumination with 270 mJ/cm^2^). We conducted a ramp from room temperature to 95 °C for over 60 min, held at 95 °C for 5 min, and cooled down again to room temperature over 60 min for the post-exposure bake. The wafer was developed for 6 min with mr-Dev 600 (micro resist technology GmbH, Berlin, Germany), followed by rinsing with 2-propanol (Technic France, Saint-Denis, France) for another 10 s and spin-drying. A hard bake with a ramp over 4 h to 180 °C, held at 180 °C for 2 h, and cool down to room temperature over 4 h was used. As a final step, the wafer was diced in two 75 mm × 25 mm microarray plates; each contained more than 1500 cavities. The individual cavities had a diameter of 300 μm. The distance between two neighboring cavities was 310 μm.

### Device Operation

We placed the microarray plate in a removable temperature-controlled holder (*T* = 37 °C) with a transparent bottom, which was mounted on a motorized XY stage (HLD117, Prior) of an inverted fluorescence microscope (Olympus IX73). We carried out the experiments in an oil bath to reduce droplet evaporation. The plate holder was filled with ∼4 mL of 1:1 (v/v) hexadecane:squalane without phospholipids and 50 μL of water in all four edges to reduce droplet shrinkage. We used a self-made microfluidic T-junction device made of polycarbonate (PC) to generate droplets of ∼25 nL by continuously injecting the aqueous phase (flow rate: 0.5 μL/min) into the immiscible oil phase (flow rate: 2 μL/min). The droplets were transported through a capillary onto the microarray plate. In this time, the phospholipids in the oil aligned along the water/oil interface and formed a monolayer.^27,28^ We mounted the end of the capillary on a motorized Z stage (M-403.2PD, Physik Instrumente, Karlsruhe, Germany). The generated droplets were detected in the capillary holder with a custom-made optical droplet detection system.^22^ This was utilized to selectively deposit a single droplet per predefined position by synchronizing droplet generation and capillary and microarray movements. All components of the microscope and the capillary were controlled by the software YouScope for automated microscopy (R2018, v2.1.0).^29^

### Image Acquisition and Analysis

Fluorescence and bright-field pictures were recorded using a light source (Lumen 300, Prior and TH4-200, Olympus) and a complementary metal oxide semiconductor (CMOS) camera (Zyla 4.2, Andor) connected to the Olympus IX73 microscope. To track fluorescein, calcein, and PEG4-NBD, a blue excitation filter set (exciter HQ470/40x, dichroic 500dcxr BS, and emitter E515lpv2; Chroma Technology Corp, Bellows Falls, VT, U.S.A.) was used. For rhodamine 6G, a green excitation filter set (exciter 525/50m, dichroic Q565lp, and emitter 588 LP; Chroma Technology Corp.) was used. To minimize environmental influences and heat exchange, a black anodized lid was placed on top of the plate holder. The bright-field images were used to determine the diameter of the DIB.

The recorded fluorescence signals were evaluated using Fiji^30^ and OriginPro (2019, 9.6, OriginLab Corporation). First, we subtracted the background fluorescence for data evaluation. In a next step, we normalized the fluorescence to compensate for potential leakage into the oil phase, membrane partitioning, shrinkage of droplets, or fluorescence bleaching. For the normalization, the sum of fluorescence of all droplets in the first image was set to 100%. For the subsequent images, fluorescence of all droplets was normalized proportionally to keep a constant total signal over time. Donor and acceptor droplets spotted on adjacent cavities, which did not touch each other and therefore did not form a DIB, were excluded.

### Combination of the Droplet Spotter with LC–MS

For subsequent LC–MS analysis, we used a recently developed protocol to split the droplet pairs.^24^ The capillary used before for spotting of droplets was flushed with hexadecane/squalane (1:1) and placed between two connecting droplets to separate the droplet pairs. To extract individual droplets, the capillary was flushed with fluorinated oil (HFE-7500, 3 M Novec, Hadfield, U.K.) and connected to a 50 μL glass syringe (Hamilton, Switzerland). The center of the capillary was lowered to a height where it slightly squeezed the droplet. Subsequently, by slowly pulling the glass syringe, the droplet was aspirated. Afterward, the capillary was moved over a small tube (TreffLab Degersheim, Switzerland), and the aspirated droplet was ejected. Following this, the tube was centrifuged (3300 rounds per minute, MiniSpin, Eppendorf) to assure that the droplet moved to the bottom. The tube was then heated to 60 °C for 30 min to induce water/solvent evaporation. After this step, the samples were redissolved in LC–MS grade water/acetonitrile (2:1) (HiPerSolv Chromanorm, VWR).

The sample was analyzed in a 1260 II Infinity LC Quaternary system coupled to a single quadrupole atmospheric pressure ionization-electrospray (API-ES) G6130B mass spectrometer (Agilent). It was controlled through the Agilent OpenLAB CSD ChemStation (C.01.08). To separate the analytes, we used a reversed-phase 100 mm Poroshell 120 SB-C8 column (Agilent) and the solvents acetonitrile, LC–MS grade water, and isopropanol (hypergrade for LC–MS, Merck, Germany) together with 5 mM ammonium formate (AF, Agilent) buffer. All analytes were detected with selected ion monitoring (SIM) in a positive mode. The signal was obtained by integration of the peak area in the SIM MS spectra.

### Permeability Calculations

In our fluorophore permeation studies, we were able to image the permeation process over a long time period. Equations 1 and 2 allow for extracting kinetic rate constants by plotting the mass of the model permeant in the acceptor (*M*_a_) and donor droplets (*M*_d_) over time (*t*). The rate constants (*k*) describe the fraction of the model permeant that is transferred over the barrier per time (mass transfer rate constants). As the permeation is equilibrative, the observed kinetics, *i*.*e*., change in fluorescence over time, are dependent on both *k*_da_ (donor to acceptor) and *k*_ad_ (acceptor to donor). As long as the fluorescence intensity is proportional to the concentration of the fluorophore, the intensity can be directly plotted and analyzed.^31^
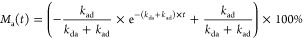
1
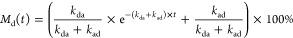
2

Equations 1 and 2 were fitted to the data with OriginPro. The iteration algorithm used was Levenberg Marquardt. Data from every droplet pair were individually fitted and evaluated. In the graphs, we show the fit for the mean values. The rate constant for the transport from the donor to acceptor compartment (*k*_da_) can be converted into the apparent permeability coefficient (*P*_app_).^31^ We used eq 3 for the calculation of *P*_app_ from the fitted *k*_da_ (*P*_app,fit_) under the assumption that volumes of the two compartments were equal (*V*_d_). We further approximated that the area of the droplet interface bilayer is circular,^32^ and we calculated the area (*A*) for the individual droplet pairs via the measured droplet interface bilayer diameter from the bright-field images (as indicated in Table S1).
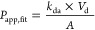
3

To determine the *P*_app_ from MS measurements with fewer time points, we used eq 4. *M*_d_(0) is the sample mass in the donor droplet at the start. Δ*M*_a_ represents the sample mass permeated into the acceptor droplet after a given time (Δ*t*). We differentiated between sink and nonsink conditions.^31,33^ Under sink conditions, the transfer of substances back from the acceptor to the donor compartment can be neglected. We assumed approximately constant Δ*M*_a_/Δ*t* as long as less than 10% of the initial donor content permeated into the acceptor compartment.
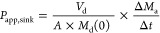
4

### Permeation Kinetics with Droplets in Series

For several acceptor droplets in series, the numerical solutions of a differential equation system according to eqs 5 to 7 were fitted to the normalized fluorescence data of donor and acceptor droplets. The solutions of the differential equation system and the fitting were performed with the Matlab (version 2018a, Mathworks) functions ode15s, fmincon (minimizing the sum of squared residues), and MultiStart.
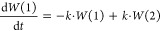
5
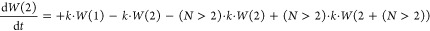
6
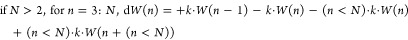
7

*W*(*n*) is the fluorescence in the water phase of droplet *n*, *k* is the mass transfer rate constant (the fit parameter), and *N* is the total number of donor and acceptor droplets. The expression *N* > 2 equals 1 if *N* > 2 and 0 if *N* ≤ 2, and *n* < *N* equals 1 if *n* < *N* and 0 if *n* ≥ *N*.

## Result and Discussion

### Spotting Platform for DIB Formation

**Figure 1:**
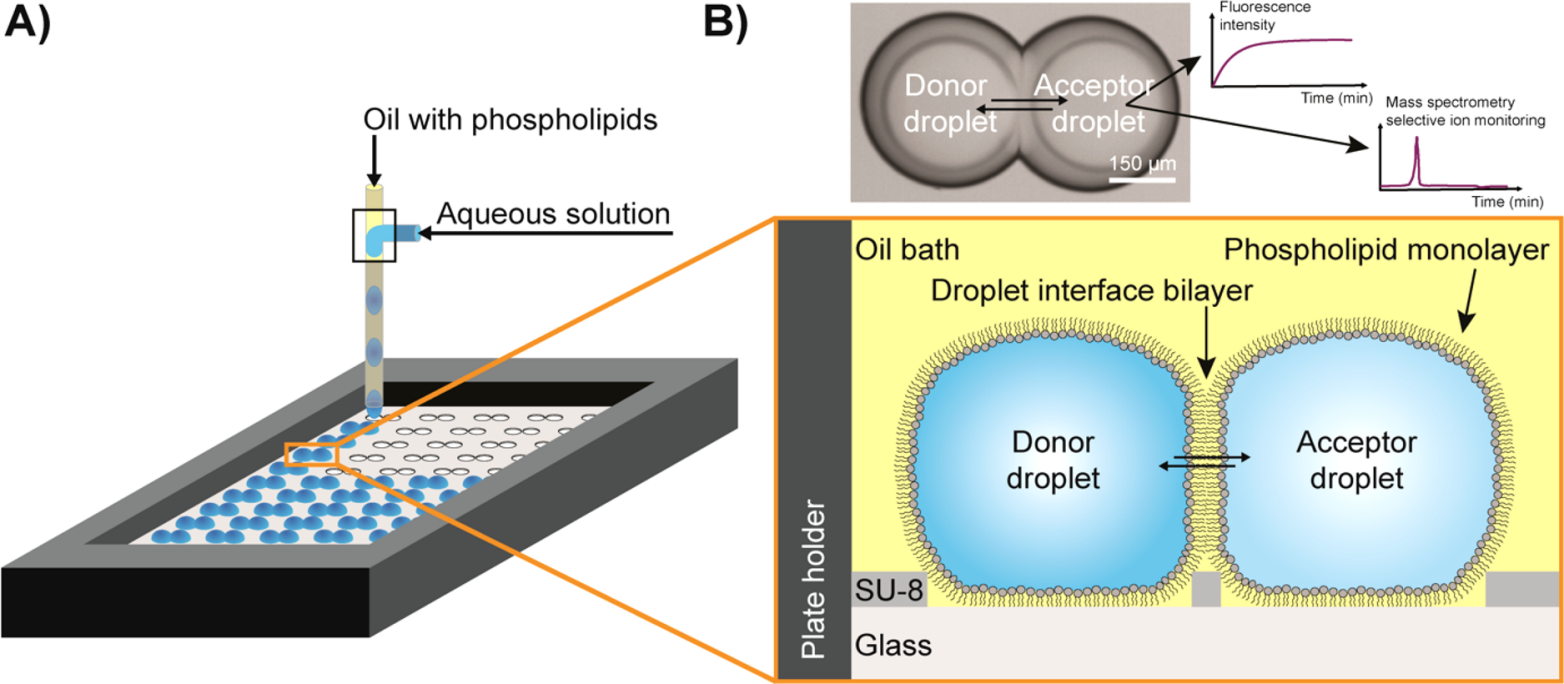
Creation of droplet interface bilayers (DIBs) for permeation studies. (A) Creation and positioning of droplets on an open platform. Nanoliter droplets are formed at a microfluidic T-junction from an aqueous solution containing the model permeant and oil with phospholipids. The droplets are deposited on a glass plate into cavities, which are built in a layer of the photoresist SU-8. The enlarged schematic depicts the side view. Donor droplets with model permeants are spotted next to the acceptor droplets. The DIB forms in between these droplet pairs and facilitates passive permeation of compounds from the donor to acceptor droplet and vice versa. (B) Micrograph showing a droplet pair with the DIB. The small circles inside the droplets are shadows of the cavities. We measure either the fluorescence intensity in the donor and acceptor droplets or take mass spectra of the droplet contents.

We developed a microfluidic platform with the aim to understand key parameters of membrane permeation of molecules (Figure 1). Aqueous droplets were created in a simple microfluidic T-junction and afterward were positioned on hydrophilic cavities on the surface of a plate. First, the donor droplets were deposited, containing the permeating compound, and in the second spotting procedure, the acceptor droplets were added to the cavities in close proximity of the donor droplets (Video S1 in the Supporting Information). Droplets hosted by neighboring cavities contacted each other. As the droplet–oil interfaces consisted of a self-assembled phospholipid monolayer, the contact between this aqueous droplet and another droplet united the lipid monolayers, creating the DIB of roughly 5 nm thickness.^4,34^ The droplets were covered by oil to prevent evaporation. The oil bath had a volume of ∼4 mL and did not contain phospholipids. Therefore, we expected that the few remaining phospholipids, which did not assemble to the monolayer of the aqueous droplets after droplet formation, were quickly diluted in the large volume of the oil bath. These diluted phospholipids were assumed to assemble on the surface of the four 50 μL water reservoirs at the edges of the plate holder. One 50 μL water reservoir had more than a 150 times larger area compared to the ∼25 nL droplet. The ∼25 nL droplets were already saturated with phospholipids when spotted on the plate, whereas the 50 μL water reservoirs did not contain any phospholipids at the water/oil interface in the beginning of the experiment. It is also possible that the diluted phospholipids are remaining in the oil bath or assemble at the oil-air interface.

After formation of a DIB within less than 1 min, we monitored the permeation process. Previously, we proved the formation of unilamellar DIBs by forming alpha-hemolysin pores with our method.^24^ DIB membranes that could incorporate transmembrane proteins or toxins behaved like “oil-free” membrane structures, such as vesicles.^24,35^ Even when a tiny amount of oil was still present in the membrane, it should not affect our measurement since the membrane of a biological cell also contains cholesterol precursors. The current size of the droplets can be reduced with other methods than the used T-junction to sizes of mammalian cells (15–20 μm). Furthermore, the spotting plate is scalable and could be fabricated with smaller droplet deposition sites (theoretically down the resolution of photolithography, i.e., ∼1 to 2 μm). The number of deposited droplet pairs and networks could be increased up to several hundred thousands.

### Fluorophore Permeation Analysis with Symmetric Membranes

**Figure 2:**
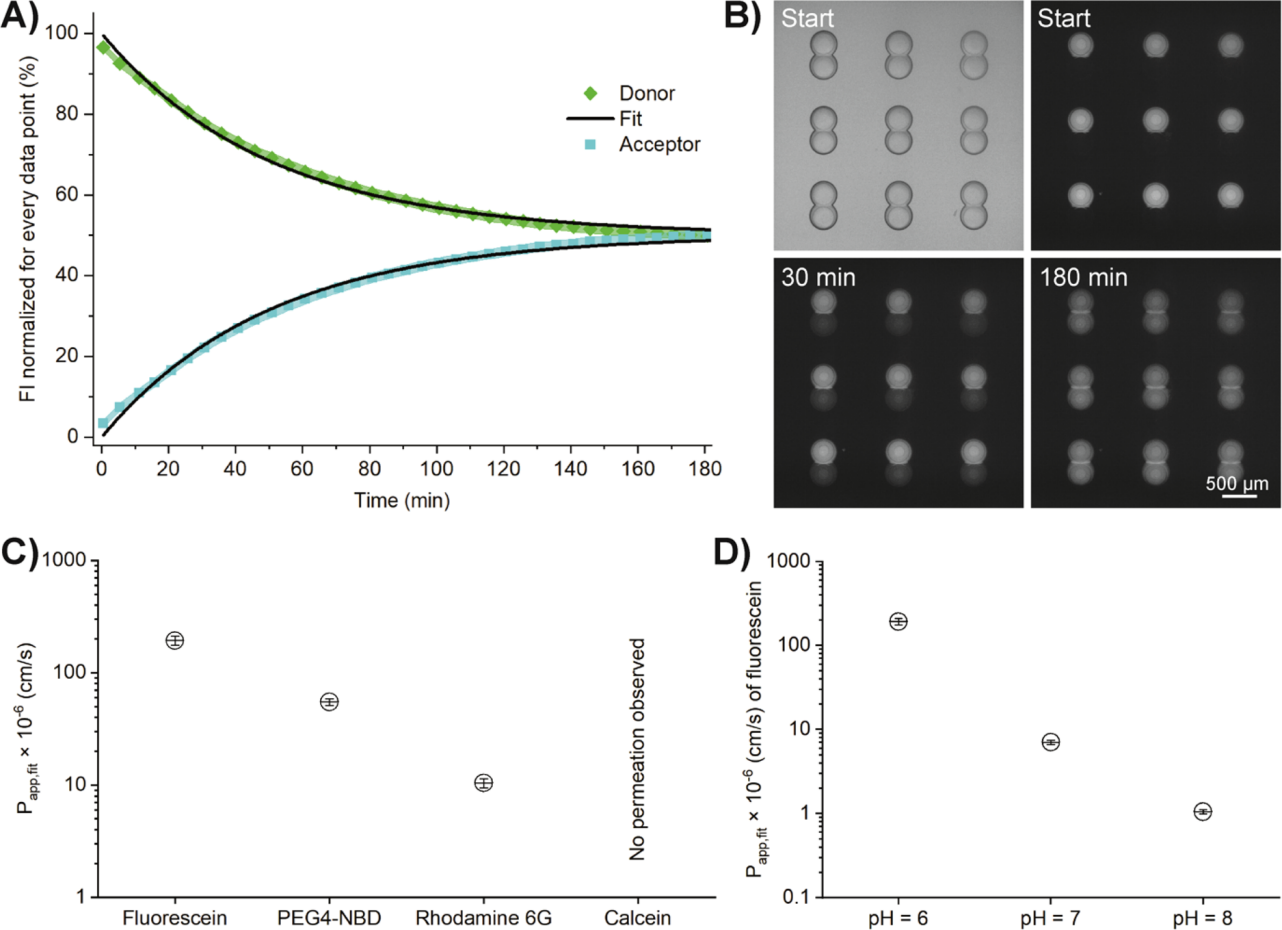
Permeation of different fluorophores across 100% DOPC droplet interface bilayer (DIB) membranes. (A) Mean normalized fluorescence intensity (FI) over time of rhodamine 6G in donor and acceptor droplets at pH 6.0. The color-shaded areas represent the standard deviation of every data point (*N* = 27). The fit is shown for the mean values. (B) Bright-field (top left) and fluorescent images of donor and acceptor droplets for the graphs shown in panel A. (C) Apparent permeability coefficients and standard deviations of fluorescein, PEG4-NBD, rhodamine 6G, and calcein (all at pH = 6.0, *N* = 27). (D) Mean *P*_app,fit_ and standard deviations of fluorescein at different pH (*N* = 27).

We optimized and evaluated the method with fluorescent dyes (rhodamine 6G, fluorescein, PEG4-NBD, and calcein) and performed kinetic measurements to assess the respective *P*_app,fit_. We chose a pH of 6 in these experiments as approximation to the pH of the small intestine where most drugs are absorbed^36^ and set the physiological temperature at 37 °C. Figure 2A,B and Figure S1 show the permeation of rhodamine 6G from donor to acceptor droplets until an equilibrium is reached. Likewise, PEG4-NBD and fluorescein permeated across the DIB (Figures S2 and S3), while no permeation was observed for calcein (Figure S4). This observation is in good agreement with the very low permeability coefficients (10^–10^ to 10^–11^ cm/s) reported for calcein permeation across liposome membranes.^37^ For isolated droplets that contain one of these fluorophores but are not connected to an acceptor droplet, the fluorescence intensity is constant, and therefore, we assume to have no leakage of permeants into the oil phase. Likewise, isolated acceptor droplets deposited near donor droplets do not exhibit a fluorescent signal over time (Figures S1–S3).

The apparent permeability coefficients were determined using eqs 1–3 (Figure 2C); all fitting values are listed in the Supporting Information, Table S1. In addition, we determined the *P*_app,fit_ of fluorescein at pH 6, 7, and 8 (Figure 2D and Supporting Information Figures S3, S5, and S6). As expected, the *P*_app,fit_ of fluorescein dropped significantly for higher pH values, since the phenolic p*K*_a_ of fluorescein is 6.4, and the fraction of the di-anionic form (carboxylate and phenolate) increases from pH 6 to 8, resulting in reduced permeability.

The *P*_app,fit_ values for fluorescein across 100% DOPC DIBs match well with findings in former studies with DIBs,^4,12^ vesicles,^26^ or cell monolayers.^38,39^ For example, our values at pH 6, 7, and 8 were (193.03 ± 17.79) × 10^–6^, (7.05 ± 0.42) × 10^–6^, and (1.05 ± 0.06) × 10^–6^ cm/s, respectively, similar to the permeation constants reported by Schlicht and Zagnoni ((2.01 ± 1.46) × 10^–6^ cm/s at pH 7.4),^12^ and by Nisisako et al. ((60.0 ± 22.4) × 10^–6^ cm/s at pH 6.4 and (5.1 ± 1.8) × 10^–6^ cm/s at pH 7.5).^4^ Likewise, the determined permeability constants for PEG4-NBD and rhodamine 6G are in the same order of magnitude to previous results.^26,40^

### Fluorophore Permeation Analysis with Asymmetric Membranes

**Figure 3:**
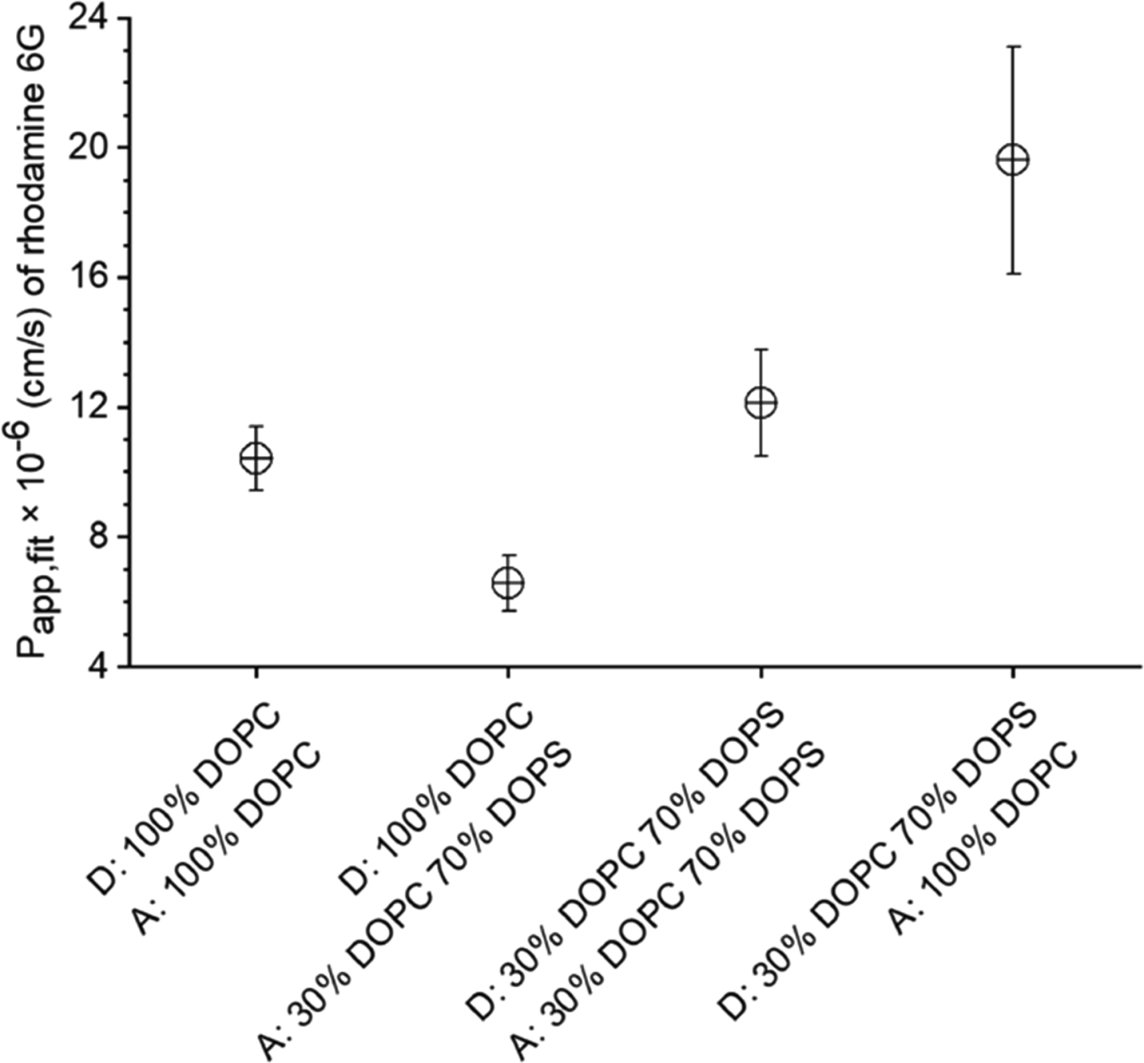
*P*_app,fit_ for rhodamine 6G at pH 6.0 across symmetric and asymmetric DIBs. The error bars represent the standard deviation, *N* = 27 droplet pairs.

Next, we varied the composition of the DIB. We created symmetric DIBs by spotting droplet pairs of the same type, e.g., all with a DOPC monolayer, and asymmetric DIBs by spotting donor and acceptor droplets with different lipid monolayers. We chose DOPC and, as a second monolayer, a formulation of 30% DOPC and 70% DOPS, because the negatively charged phosphatidylserine (PS) is an important constituent of the plasma membrane of cells. PS is located in the inner leaflet of healthy cells and transferred to the outer leaflet in apoptotic cells. The asymmetry due to PS may result in an asymmetric partitioning of the permeant between the two lipid layers. We monitored the influence of this asymmetry on the permeation kinetics of rhodamine 6G (Supporting Information, Figures S7–S9). Depending on the membrane composition, extensive lag phases were observed in the fluorescence–time curves of both donor and acceptor droplets. Analysis with eqs 1 and 2 for the complete data set, including the lag phase, resulted in different rate constants for the four membrane compositions. Taking into account the differences in DIB diameters in eq 3 still resulted in different *P*_app,fit_ values for the different membrane compositions (Figure 3 and Supporting Information Table S1). The highest apparent rate constant and *P*_app,fit_ were observed for the permeation across the asymmetric lipid bilayer of 30% DOPC and 70% DOPS to 100% DOPC (Supporting Information, Figure S7). The lowest *P*_app,fit_ and apparent rate constant for rhodamine 6G were found for the opposite composition, which also had the most prominent lag phase (Supporting Information, Figure S8). For symmetric DIBs, the values for the *P*_app,fit_ were in between those for asymmetric DIBs (Supporting Information Figure S9 and Figure 2A).

In theory, the permeability coefficients across asymmetric membranes should be independent of the direction of the initial concentration gradient.^31^ The differences between the *P*_app,fit_ values of the two asymmetric bilayers in our experiments could result from interactions of cationic rhodamine 6G with the negatively charged DOPS, visible by increased fluorescence at the lipid monolayer of the droplets. This accumulation or aggregation of the fluorophore is clearly visible in the Supporting Information Figures S8A and S9A, where the acceptor droplet’s monolayer comprises 30% DOPC and 70% DOPS. These interactions may result in the observed lag phase in the fluorescence–time curves (Supporting Information, Figures S8 and S9), reducing the apparent rate constants when fitting the complete data set with eqs 1 and 2. Further general sources of error could be leakage out of the droplet and bleaching, both reducing the observable fluorophore in the droplets over time.

It should be mentioned that the asymmetric membranes could equilibrate due to lipid flip-flop,^41^ resulting in equal lipid compositions on both sides of the DIB. Since the exchange of lipids is very slow in defect-free membranes (∼10^–15^ s^–1^),^42^ we expect a stable asymmetric DIB for several hours and assume that lipid flip-flop has no influence on our results.

### Fluorophore Permeation over Multiple Compartments

**Figure 4:**
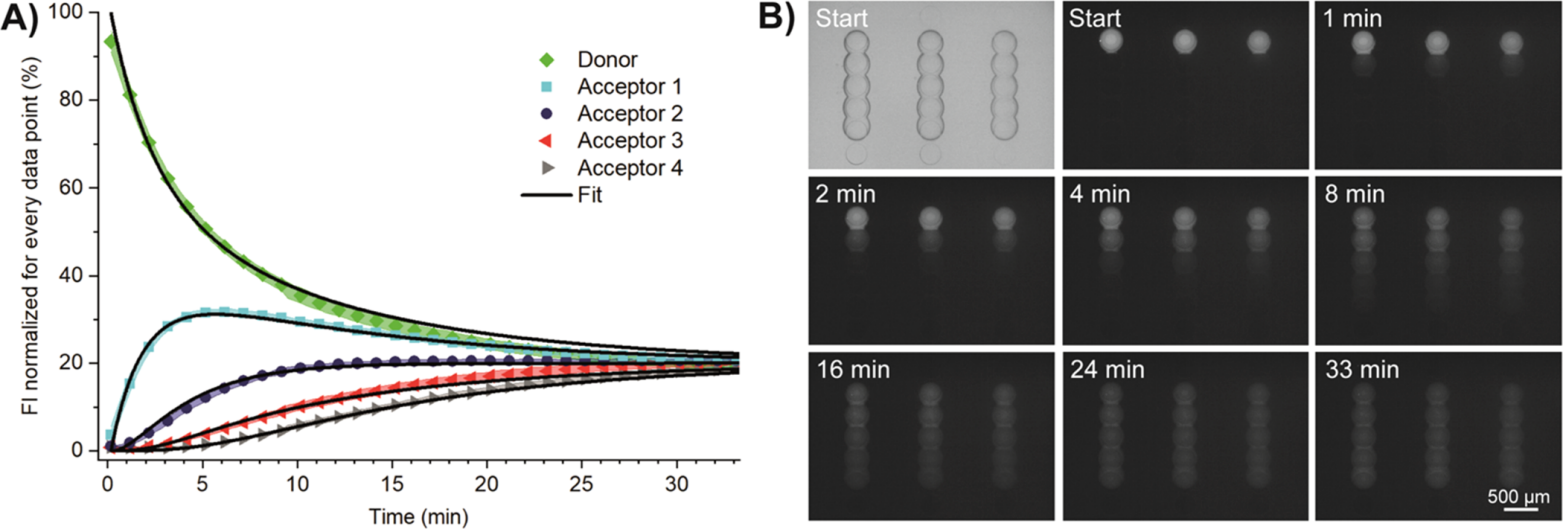
Permeation of fluorescein over multiple compartments. (A) Mean fluorescence intensity (FI) and standard deviation (color-shaded areas) over time from one donor to four acceptor droplets (100% DOPC droplet interface bilayer, pH 6.0, *N* = 12 droplet networks). Black lines: fits according to eqs 5–7. (B) Bright-field (top left) and fluorescent images of the permeation.

Our platform allows for spotting droplet lines, instead of just pairs. With this, we can monitor how permeation and diffusion of compounds progress over several compartments. For creation of multiple droplets, connected via DIBs, we used a modified glass plate, where cavities were created in lines. Several fluorophore-free acceptor droplets were deposited in this line accordingly. The last droplet contained fluorescein, which permeates over the multiple compartments. Equilibrium was reached in all five droplets after approximately 30 min at pH 6 (Figure 4). We determined the *P*_app,fit_ using eqs 5–7 and 3 to be (199.68 ± 9.73) × 10^–6^ cm/s, similar to the value determined with the droplet pairs, *i*.*e*., (193.03 ± 17.79) × 10^–6^ cm/s. Permeation at pH 7.4 was slower, which was expected from the experiments with the droplet pairs and from the ionization state of fluorescein (Supporting Information, Figure S11). The *P*_app,fit_ at pH 7.4 was (19.01 ± 1.16) × 10^–6^ cm/s, higher than determined with the droplet pairs at pH 7.0 (7.05 ± 0.42) × 10^–6^ cm/s. We did not further investigate the discrepancy at the higher pH. The lag phase observed at pH 7.0 (Supporting Information Figure S5) has a higher impact on the fit parameters in the assay with only two droplets (reducing the apparent rate constants) than with several droplets in series.

### Label-free Permeation Analysis

**Figure 5:**
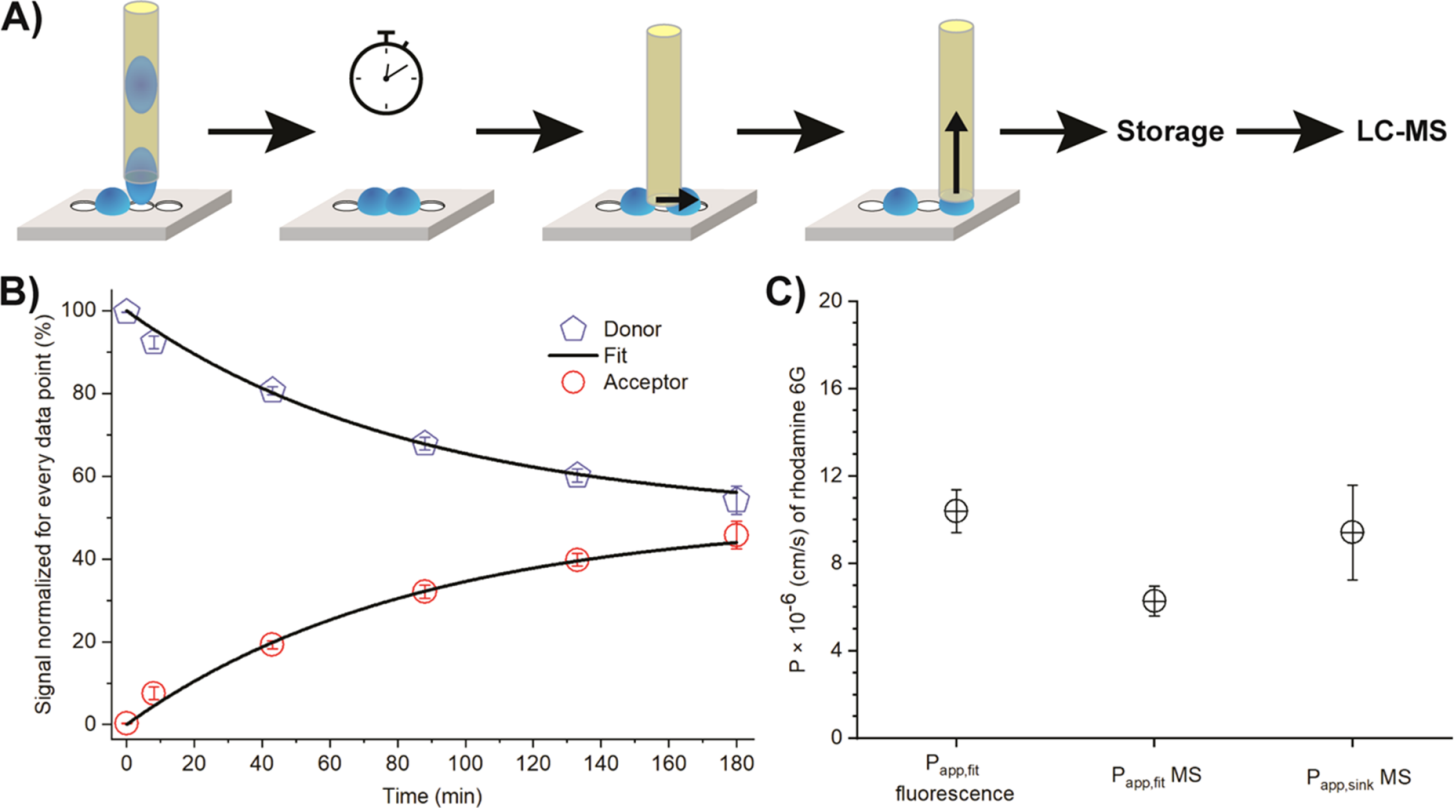
(A) Scheme of droplet separation and extraction for liquid chromatography–mass spectrometry (LC–MS) analysis. (From left to right: droplet spotting; the incubation step for allowing permeation; droplet separation; droplet aspiration; placing the droplet in a storage place to evaporate the remaining water/solvents and, following this, a defined volume of water/acetonitrile (2:1) is added for redissolution; LC–MS analysis). (B) Permeation of rhodamine 6G across droplet interface bilayer (DIB) membranes at pH 6.0 measured by MS (*N* = 4). (Black lines: fits according to eqs 1 and 2 (*P*_app,fit_). (C) Apparent permeability coefficients (*N* = 27 for *P*_app,fit_ fluorescence; *N* = 4 for MS measurements).

Finally, we developed a protocol to interface our platform with LC–MS and to measure the *P*_app_ of nonfluorescent compounds in the future. Here, we demonstrate the workflow (Figure 5A) and perform the MS analysis of donor and acceptor droplets for rhodamine 6G. In contrast to fluorescence microscopy, the analysis by MS was not done continuously but at a defined time point. For MS analysis, droplet pairs were separated by placing a capillary in between the pair (Supporting Information, Video S2), and the individual droplets were then aspirated into the capillary (Supporting Information, Video S3). These droplets were then transferred into a tube, where water was exchanged by a mixture of MS-grade water–acetonitrile. We performed this procedure for different time points and determined the MS signals (integrated peak area) for both donor and acceptor droplets (Figure 5B). We calculated the *P*_app_ by using eq 3 (*P*_app.fit_ = 6.28 ± 0.68 × 10^–6^ cm/s) as well as eq 4 (*P*_app.sink_ = 9.42 ± 2.17 × 10^–6^ cm/s using the first two data points), which assumes the sink condition (neglecting back-permeation into the donor droplet). The slightly lower value obtained when fitting all data (*P*_app.fit_ MS) can be presumably attributed to heat losses during sampling of the droplets (opening the lid positioned over the plate to allow separation and extraction of the droplet pairs). Since *P_app.sink_* MS was calculated with the droplet pairs, which were separated first, only a minimal heat loss is expected at these data points. The results confirm that our method facilitates the determination of the permeation coefficient by either fluorescent microscopy or mass spectrometry.

## Conclusions

We developed a versatile microfluidic method to determine the permeability coefficients of small molecules by monitoring their permeation across artificial lipid bilayers. The lipid bilayers are formed between two adjacent nanoliter droplets that are deposited on a glass substrate. We showed on-demand droplet generation, spotting, on-site continuous investigation of fluorescent molecules with a microscope, and subsequent LC–MS analysis, opening doors for permeability studies of nonfluorescent compounds. The method is suitable for a wide range of water-soluble and amphiphilic drugs. It may not be suitable for very lipophilic, apolar compounds with high partitioning to the oil phase, resulting in very low fluorescence in the water phase. For these compounds, the assay geometry may be modified to reach a minimal volume ratio between oil and water phases in order to be able to quantify the fluorescence in the water phase.

The method is much faster than the current state-of-the art PAMPA, i.e., normally less than 1 h for *P*_app.sink_ compared to 2–18 h of PAMPA assay time, which requires approximately 8000 times smaller compartments (*V* = 25 nL vs ∼200 μL) and can be equally parallelized as PAMPA. Therefore, it is ideally suited for rapid determination of drug permeability during the drug screening process.

Moreover, we can investigate specific aspects of the permeation process by systematic alterations of various parameters. For example, the membrane compositions can be changed and asymmetric membranes can be created, which leads to a better understanding of the permeation mechanisms. We believe that it is also possible to reconstitute membrane proteins into the DIB, which will ultimately enable studies on membrane transporters or determination of ligand–receptor binding.

## Supporting Information Available

A summary of the fitting results and microcroscopy images and graphs for various conditions (PDF)
(Video 1) The spotting process (AVI)
(Video 2) The process of splitting droplet pairs (AVI)
(Video 3) The aspiration of droplets into a capillary (AVI)

## Supplementary Material

ac0c04939_si_001.pdf

ac0c04939_si_002.avi

ac0c04939_si_003.avi

ac0c04939_si_004.avi
