# Supplementary material for: Permeation Studies across Symmetric and Asymmetric Membranes in Microdroplet Arrays
Source: Anal Chem. 2021 Mar 15;93(12):5137–44. doi: 10.1021/acs.analchem.0c04939 (PMC8014892; doi:10.1021/acs.analchem.0c04939)
Supplement: Supplementary file 1 — ac0c04939_si_001.pdf [file ac0c04939_si_001.pdf]

Supporting Information

Permeation studies across symmetric and asymmetric membranes in microdroplet arrays

Simon Bachler[1], Marion Ort[1], Stefanie D. Krämer[2], Petra S. Dittrich[1*]

[1] Department of Biosystems Science and Engineering, ETH Zurich, 4058 Basel, Switzerland

[2] Institute of Pharmaceutical Sciences, Department of Chemistry and Applied Biosciences, ETH Zurich, 8093 Zürich, Switzerland

Corresponding author, e-mail: petra.dittrich@bsse.ethz.ch

Content

Table 1: Summary of the fitting results

Figures S1 – S4:

Microscopy images and graphs for the permeation of fluorophores across symmetric DIBs

Figures S5 – S6:

Microscopy images and graphs for the permeation of fluorescein at different pH values

Figures S7 – S9:

Microscopy images and graphs for permeation across asymmetric DIBs and PS-containing DIBs.

Figures S10 – S11:

Microscopy images and graphs for the permeation across multiple compartments

Table S1: Summary of the fitting results with Equations 1 and 2, and calculation of the apparent permeability coefficient according Equation 3 for all fluorescence experiments.

Fluorophore	pH	Membrane composition		$k_{da} \times 10^{-6}$ (1/s)		$k_{ad} \times 10^{-6}$ (1/s)		~DIB diameter (µm)		$P_{app,fit} \times 10^{-6}$ (cm/s)	
		Donor	Acceptor	Mean	SD	Mean	SD	Mean	SD	Mean	SD
Fluorescein	6	100% DOPC	100% DOPC	4046.0	315.7	4046.0	315.7	259	4.7	193.03	17.79
Fluorescein	7	100% DOPC	100% DOPC	145.0	9.8	145.0	9.8	256	6.7	7.05	0.42
Fluorescein	8	100% DOPC	100% DOPC	20.3	1.2	20.3	1.2	248	3.2	1.05	0.06
PEG4-NBD	6	100% DOPC	100% DOPC	818.1	66.3	818.1	66.3	218	3.7	54.88	3.80
Rhodamine 6G	6	100% DOPC	100% DOPC	166.8	14.1	166.8	14.1	226	5.0	10.42	0.98
Rhodamine 6G	6	100% DOPC	30% DOPC 70% DOPS	99.8	13.2	99.8	13.2	220	3.7	6.56	0.85
Rhodamine 6G	6	30% DOPC 70% DOPS	30% DOPC 70% DOPS	112.8	15.1	112.8	15.1	173	16.1	12.12	1.66
Rhodamine 6G	6	30% DOPC 70% DOPS	100% DOPC	251.9	38.3	251.9	38.3	204	26.5	19.60	3.50

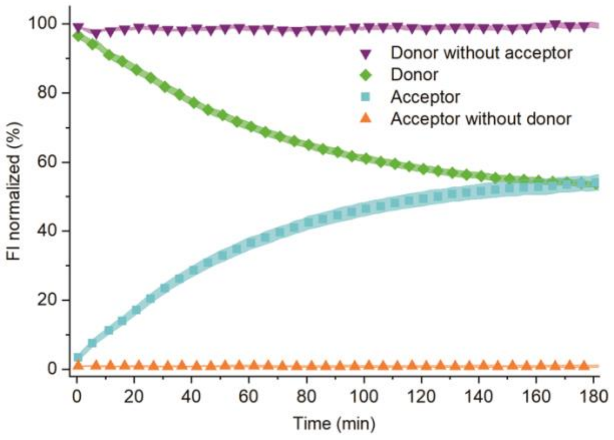

Figure S1: Permeation of rhodamine 6G across 100% DOPC DIBs at pH 6.0 and negative controls (droplets without a DIB to the adjacent droplet). Mean fluorescence intensity (FI) and standard deviation over time (N = 27 for droplet pairs and. N = 9 for the controls).

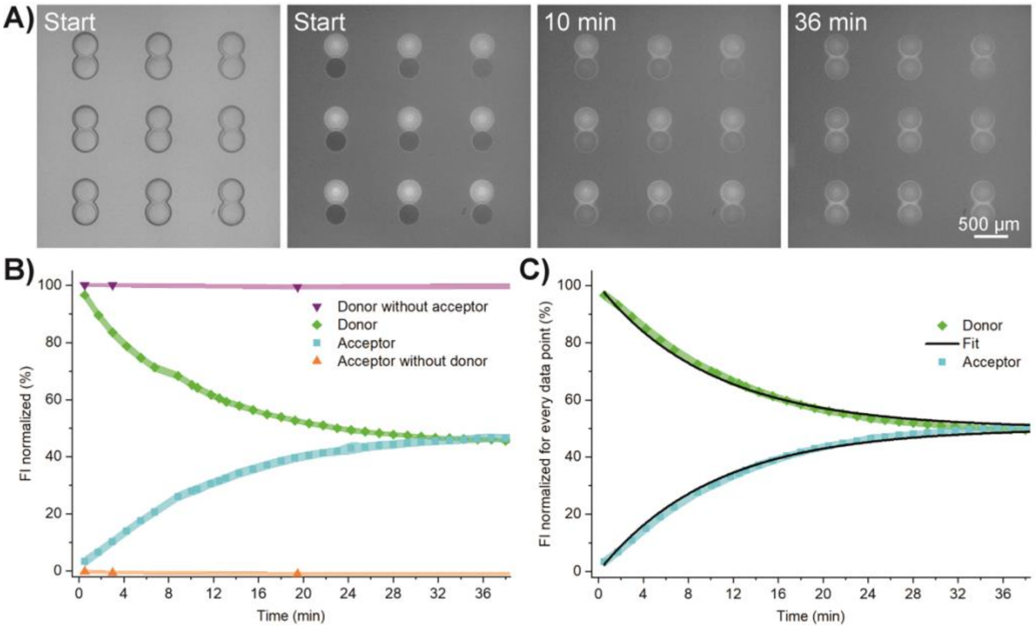

Figure S2: Permeation of PEG4-NBD across 100% DOPC DIBs at pH 6.0. A) Bright-field (top left) and fluorescent images of PEG4-NBD permeation from donor into acceptor droplets. B) Mean fluorescence intensity (FI) and standard deviations for droplet pairs and controls (N = 27 for droplet pairs and N = 9 for controls). C) In addition to the normalization to the starting fluorescence (Fig. S2B), every data point was normalized to the FI of the donor and acceptor droplets (equals 100% at the given time).

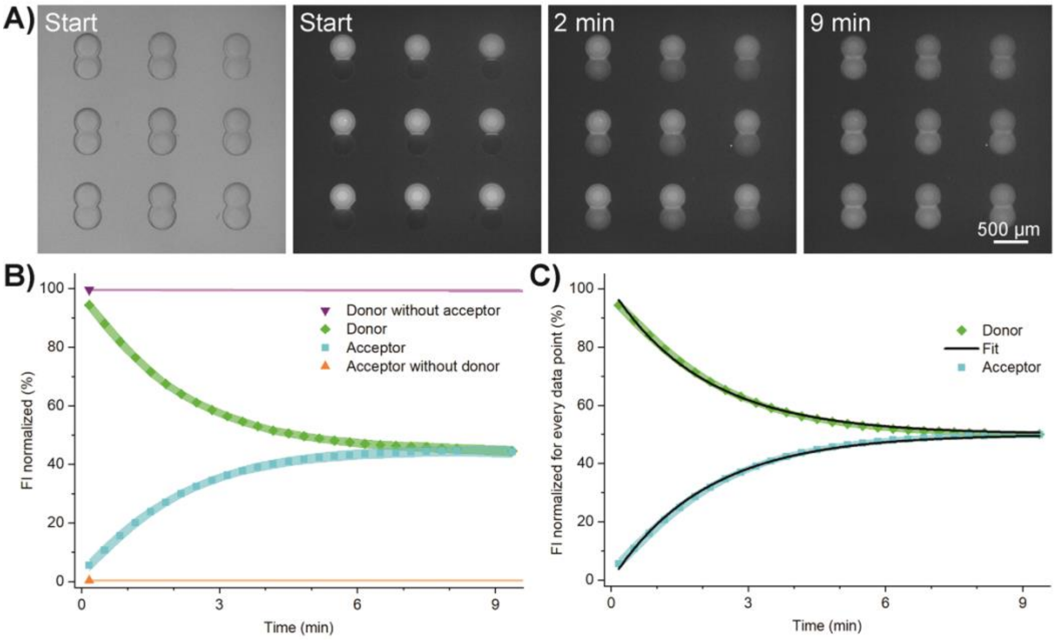

Figure S3: Permeation of fluorescein across 100% DOPC DIBs at pH 6.0. A) Bright-field (top left) and fluorescent images of fluorescein permeation from donor into acceptor droplets. B) Mean fluorescence intensity (FI) and standard deviation over time for droplet pairs and control droplets without DIB (N = 27 for droplet pairs and N = 9 for controls). C) In addition to the normalization to the starting fluorescence (Fig. S3B), every data point was normalized to the FI of the donor and acceptor droplets (equals 100% at the given time).

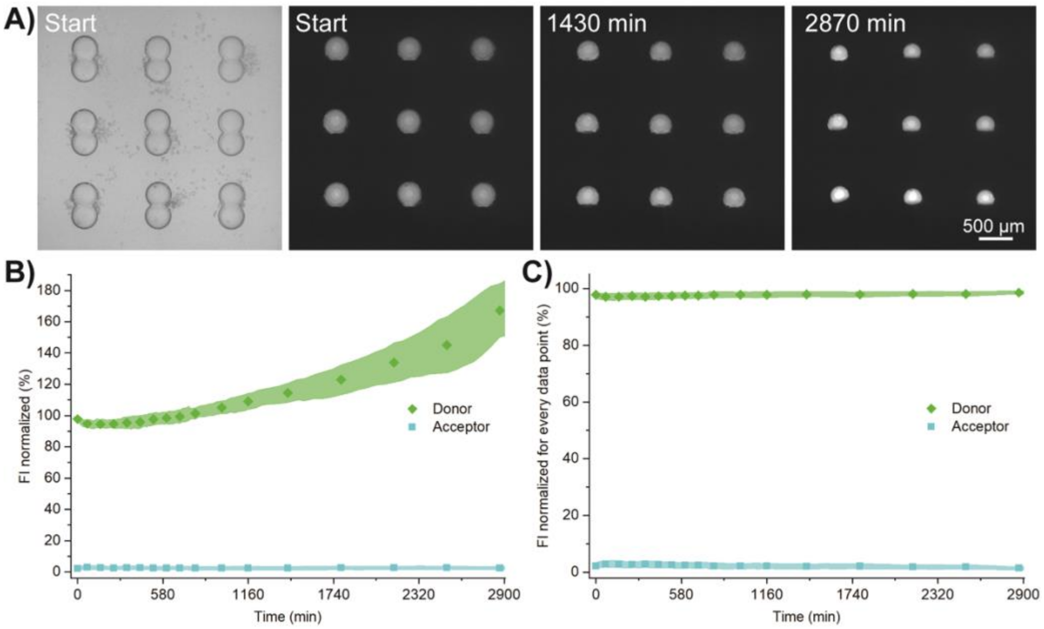

Figure S4: Calcein did not permeate across 100% DOPC DIBs at pH 6.0. A) Bright-field (top left) and fluorescent images of droplet pairs, where the donor droplet is filled with calcein. B) Mean fluorescence intensity (FI) and standard deviation over time of calcein in donor and acceptor droplets. Note the observation time of 2900 minutes (2 days), where no calcein was observable in the acceptor droplet. The increase of FI in the donor droplet is due to droplet shrinkage, i.e. water dissipates into the oil phase (N = 27 droplet pairs). C) In addition to the normalization to the starting fluorescence (Fig. S4B), every data point was normalized to the FI of the donor and acceptor droplets (equals 100% at the given time). Fitting was not possible in this experiment.

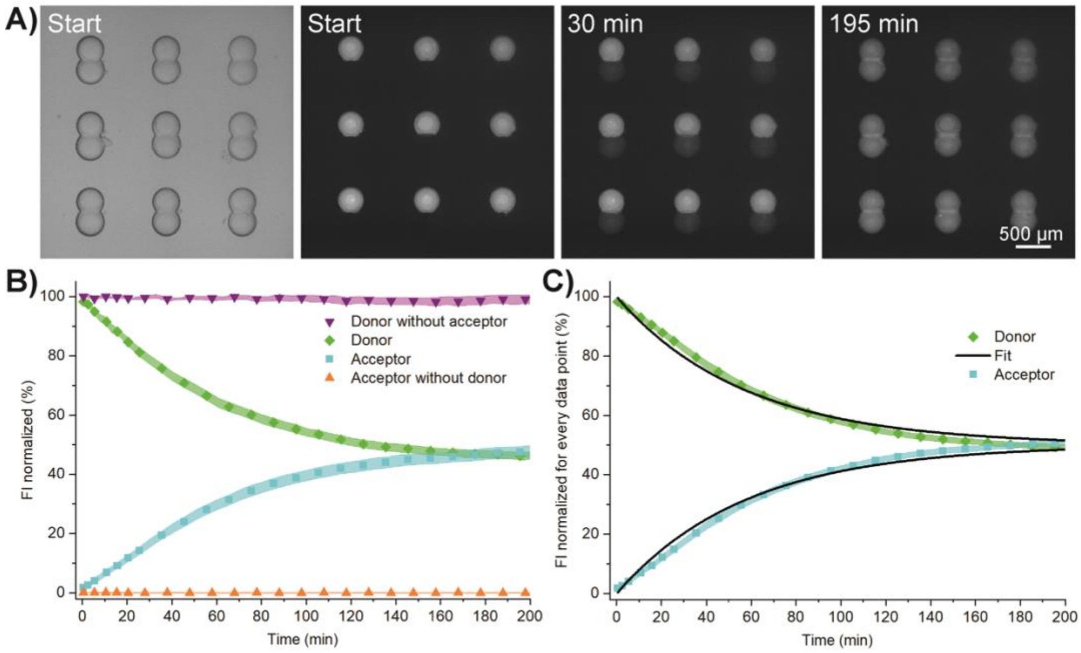

Figure S5: Permeation of fluorescein across 100% DOPC DIBs at pH 7.0. A) Bright-field (top left) and fluorescent images of fluorescein permeation from donor into an acceptor droplets. B) Mean fluorescence intensity (FI) and standard deviations over time for connected droplet pairs and isolated droplets (controls) (N = 27 for droplet pairs N = 9 for controls). C) In addition to the normalization to the starting fluorescence (Fig. S5B), every data point was normalized to the FI of the donor and acceptor droplets (equals 100% at the given time).

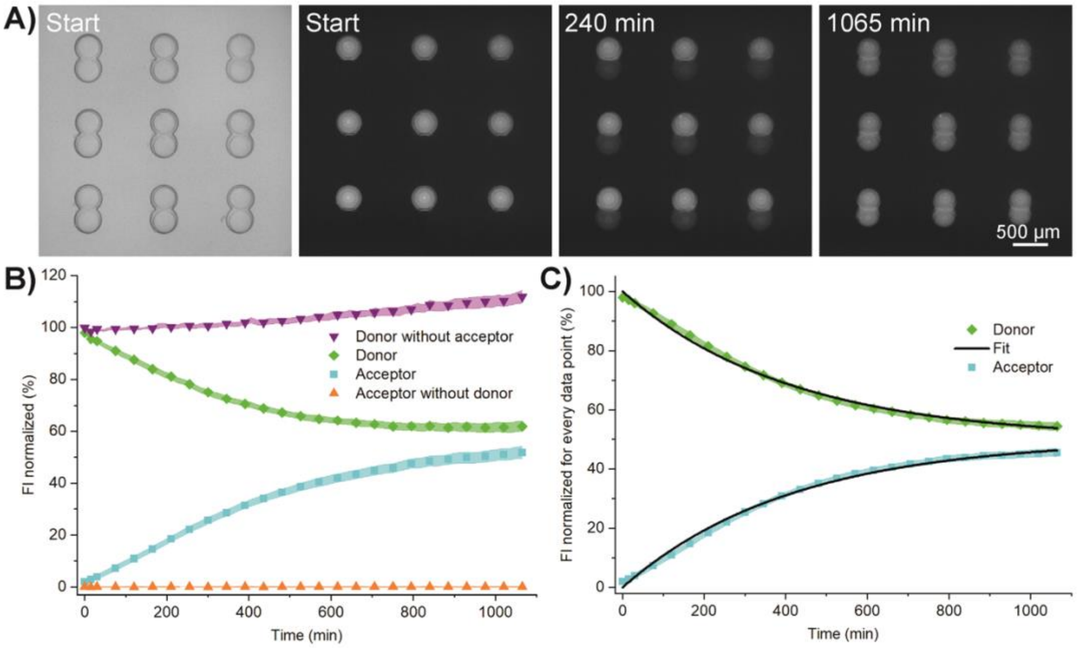

Figure S6: Permeation of fluorescein as in figure S5, except here at pH = 8.0. Note the slow permeation in figure 6B. Here, the increase in FI over time is observed due to slow droplet shrinkage.

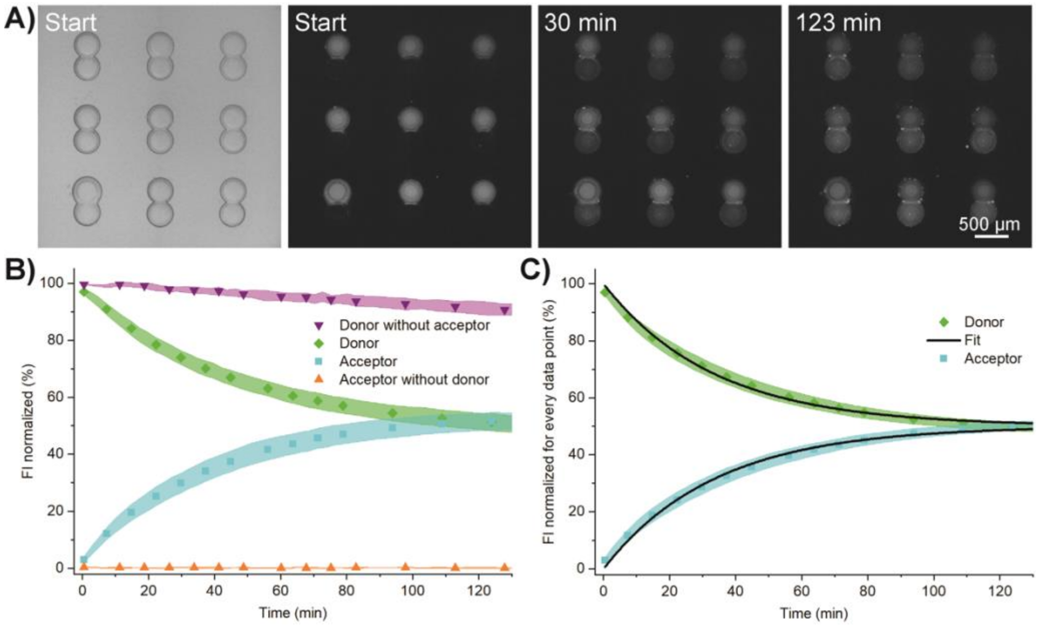

Figure S7: Permeation of rhodamine 6G across asymmetric DIBs at pH 6.0, here lipid monolayer of donor droplet: 30% DOPC 70% DOPS; and lipid monolayer of acceptor droplet: 100% DOPC. A) Bright-field (top left) and fluorescent images of rhodamine 6G permeation from donor into acceptor droplets. B) Mean fluorescence intensity (FI) and standard deviations over time of rhodamine 6G in connected droplets and controls of isolated donor and acceptor droplets, respectively (N = 27 for droplet pairs and N = 9 for controls). C) Same data as in S7B, but every data point was normalized to the FI of the donor and acceptor droplets (equals 100% at the given time).

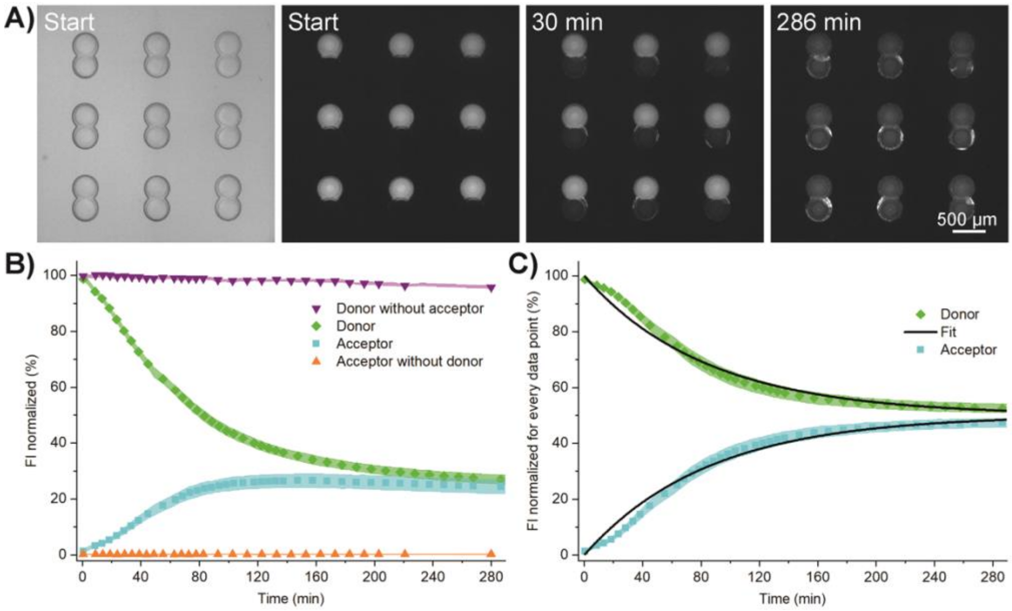

Figure S8: Same as before. The lipid monolayer of donor droplets consists of 100% DOPC and lipid monolayer of acceptor droplets of 30% DOPC 70% DOPS.

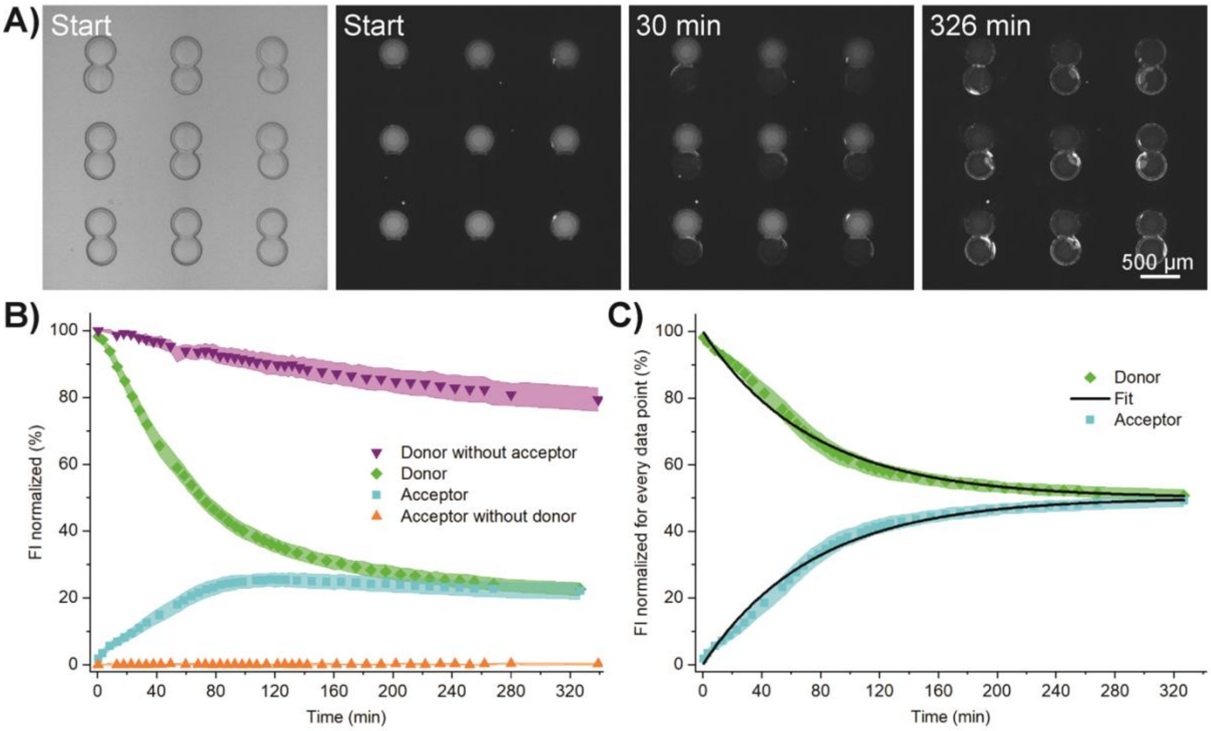

Figure S9: Permeation of rhodamine 6G across a symmetric DIB at pH 6.0 composed of 30% DOPC 70% DOPS. A) Bright-field (top left) and fluorescent images of rhodamine 6G permeation from donor into acceptor droplets. B) Mean fluorescence intensity (FI) and standard deviations over time for droplet pairs and individual, non-connected droplets filled with rhodamine 6G (“donor without acceptor”) or buffer only (“acceptor without donor”) (N =

27 for droplet pairs and N = 9 for controls). C) Same data as in S9B, but every data point was normalized to the FI of the donor and acceptor droplets (equals 100% at the given time).

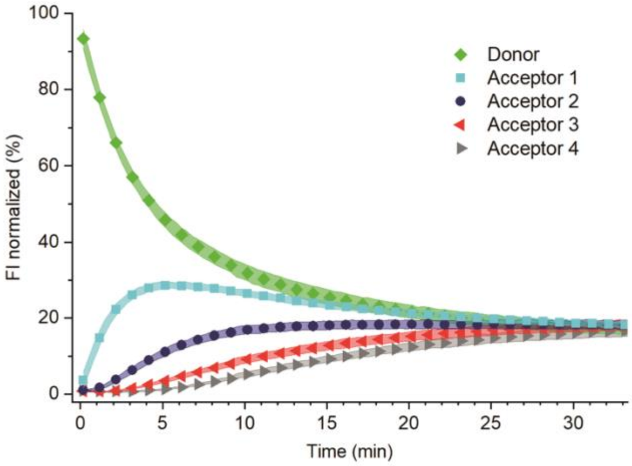

Figure S10: Permeation of fluorescein across 100 % DOPC DIBs over multiple compartments (all at pH 6.0), which are arranged in a line. Mean fluorescence intensity (FI) and standard deviations over time for fluorescein from one donor to four acceptor droplets (N = 12 droplet networks).

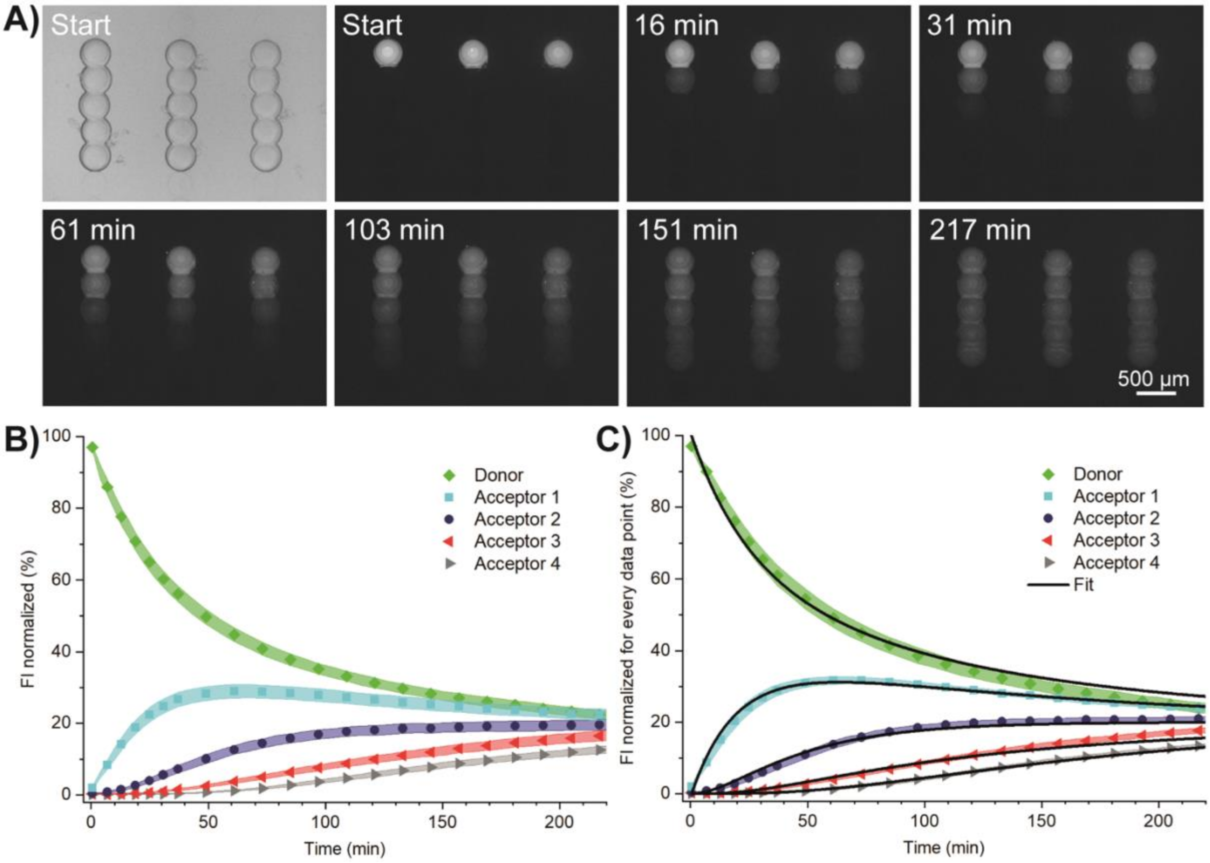

Figure S11: Permeation of fluorescein over multiple compartments at pH 7.4. A) Bright-field (top left) and fluorescent images of fluorescein permeation from one donor droplet into four acceptor droplets. B) Mean fluorescence intensity (FI) and standard deviations over time (N =

12 droplet networks). C) Same data as in Figure S11B, but every data point was normalized to the FI of the donor and acceptor droplets (equals 100% at the given time). Black lines, fit according to Equations 5-7 in the main manuscript.

Videos

Video S1: Droplet spotting

Video S2: Droplet separation

Video S3: Droplet aspiration
